# Refined Ordovician timescale reveals no link between asteroid breakup and biodiversification

**DOI:** 10.1038/ncomms14066

**Published:** 2017-01-24

**Authors:** A. Lindskog, M. M. Costa, C.M.Ø. Rasmussen, J. N. Connelly, M. E. Eriksson

**Affiliations:** 1 Department of Geology, Lund University, Sölvegatan 12, SE-223 62 Lund, Sweden; 2 Natural History Museum of Denmark, University of Copenhagen, Øster Voldgade 5–7, DK-1350 Copenhagen K, Denmark; 3 Centre for Star and Planet Formation, University of Copenhagen, Øster Voldgade 5–7, DK-1350 Copenhagen K, Denmark; 4 Center for Macroecology, Evolution and Climate, University of Copenhagen, Denmark

## Abstract

The catastrophic disruption of the L chondrite parent body in the asteroid belt c. 470 Ma initiated a prolonged meteorite bombardment of Earth that started in the Ordovician and continues today. Abundant L chondrite meteorites in Middle Ordovician strata have been interpreted to be the consequence of the asteroid breakup event. Here we report a zircon U-Pb date of 467.50±0.28 Ma from a distinct bed within the meteorite-bearing interval of southern Sweden that, combined with published cosmic-ray exposure ages of co-occurring meteoritic material, provides a precise age for the L chondrite breakup at 468.0±0.3 Ma. The new zircon date requires significant revision of the Ordovician timescale that has implications for the understanding of the astrogeobiologic development during this period. It has been suggested that the Middle Ordovician meteorite bombardment played a crucial role in the Great Ordovician Biodiversification Event, but this study shows that the two phenomena were unrelated.

## Abstract (web summary)

The high amount of L-type chondrites discovered in Ordovician sediments has previously been linked with the Great Ordovician Biodiversification Event. But here, Lindskog *et al*. present new zircon ages that date the chondrite dispersion to 468.0±0.3 Ma, showing that the two events may be unrelated.

L-type ordinary chondrites represent the largest group of meteorites striking Earth with a record of arrival extending from the Ordovician to today^1^,^2^. A common resetting of the ^40^Ar/^39^Ar chronometer in recently fallen meteorites at 470±6 Ma has been interpreted to reflect the breakup of the L chondrite parent body^3^. This event has left an unusual mark in the sedimentary record on Earth through a drastic increase in L-chondritic matter in Middle Ordovician strata. Most notably, more than one hundred macroscopic fossil meteorites (>95% of all pre-Quaternary specimens known) have presently been found and all but one are identified as L chondrites. The meteorite-bearing strata have been shown to contain very high concentrations of sand-sized chromite (chromium-iron oxide mineral) grains with typical L-chondritic chemical composition that appear to signal an enhanced influx of micrometeorites to Earth in the wake of the L chondrite breakup event^2^,^4^.

The meteorite-bearing strata overlap with an important phase of the so-called Great Ordovician Biodiversification Event (GOBE), during which the global marine biota diversified extensively and new ecologic niches were established^5^,^6^,^7^,^8^,^9^. More specifically, the arrival of the first known meteorites in the wake of the L chondrite breakup coincides with a significant increase in brachiopod diversity in Baltoscandia. This observation has led to the suggestion of a causal link between the arrival of L chondrite meteorites after the breakup of their parent body and the onset of the GOBE, implying that the effects of the meteorite bombardment on Earth would have forced biodiversification processes^10^.

Here we present new U-Pb age data from recently discovered and well-preserved zircon grains from a distinct bed within the meteorite-bearing interval in Sweden. Using a novel approach, the U-Pb data is combined with published cosmic-ray exposure (CRE) ages of co-occurring meteoritic material to pinpoint the timing of the L chondrite breakup event while simultaneously refining the Ordovician timescale. This method holds promise to be used as an independent geochronologic calibration tool for much of the Ordovician, and possibly beyond.

## Results

### Geologic setting

**Figure 1:**
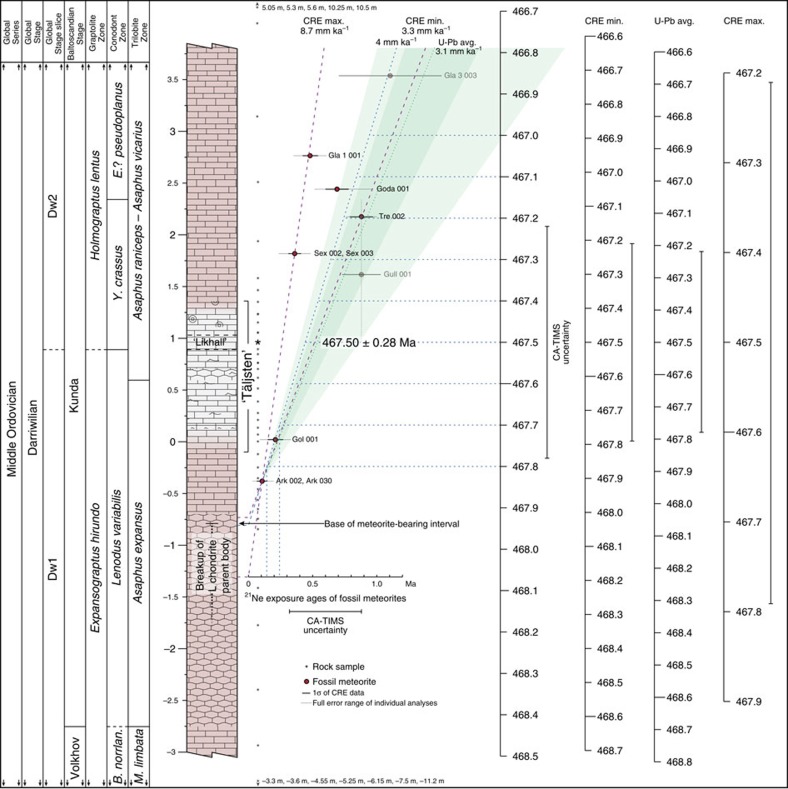
Stratigraphic and geochronologic framework. Stratigraphic column^15^, with the stratigraphic positions and CRE ages of fossil meteorites indicated in the diagram to the right^18^,^19^,^21^. Stippled purple lines indicate maximum (CRE Max.) and minimum (CRE Min.) sedimentation rate provided by the CRE data. A middle line (blue) corresponding to a sedimentation rate of 4 mm ka^−1^ is included to illustrate how the CRE data can be translated into a high-resolution timescale. The green-shaded field indicates long-term time development calculated from U-Pb data (dark=central dates of datum points; light=incl. uncertainties), with dotted line (green) indicating average^23^. Timescales resulting from the CRE Max. and CRE Min. lines are illustrated to the right in the figure, together with a timescale produced from the average sedimentation rate indicated by U-Pb data. Asterisks indicate levels searched for zircon.

A shallow epeiric sea covered large parts of Sweden and the surrounding Baltoscandian region during much of the early Palaeozoic. This widespread inundation together with low-relief (peneplaned) landmasses resulted in restricted weathering and, consequently, extremely limited siliciclastic input to the basin. Net sedimentation rates were typically on the order of millimetres per millennium^11^. The table mountain Kinnekulle in southern Sweden preserves a near-complete succession of Cambrian through lower Silurian marine sedimentary rocks^12^. During most of the Ordovician, this area was situated several hundreds of kilometres from the mainland. The depositional environment at this time was tectonically stable, a condition that has generally persisted throughout the ages. ‘Orthoceratite limestone', a condensed cool-water carbonate facies, forms the main rock type in the Middle Ordovician interval. These strata are typically brownish to rusty-red in colour, but a temporary change into grey limestone occurs in the Kunda Baltoscandian Stage, which correlates with the lower-middle Darriwilian (Fig. 1). This c. 1.5-m-thick grey interval, which by quarry tradition is known as the ‘Täljsten' (‘Carving stone'), forms an important marker level in the regional stratigraphy. The ‘Täljsten' is associated with widespread changes in the depositional environment and increased biodiversity, due to a significant shallowing in sea level^13^,^14^. The active Thorsberg quarry of eastern Kinnekulle (WGS84 58°34′45″N, 13°25′46″E) hosts a c. 6-m-thick succession of rocks that includes the ‘Täljsten'^15^. This quarry is the main source of macroscopic fossil meteorites^2^. The lowest stratigraphic horizon known to contain meteorites lies c. 0.5 m below the base of the ‘Täljsten', and the highest occurrence lies c. 4 m above this level (Fig. 1).

### The age of the meteorite-bearing strata

**Figure 2:**
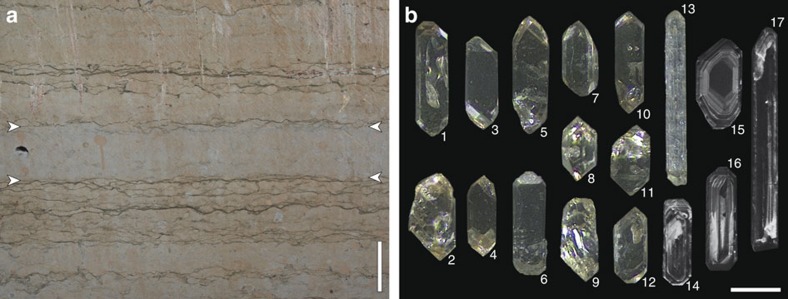
The zircon-bearing bed and zircon grains. (**a**) The ‘Likhall' bed (bracketed between arrows), as seen at the Thorsberg quarry. The scale bar corresponds to 0.1 m. (**b**) Representative zircon grains, illustrating external (1–13, reflected light) and internal (14–17, cathodoluminescence) characteristics. The scale bar corresponds to 100 μm.

**Figure 3:**
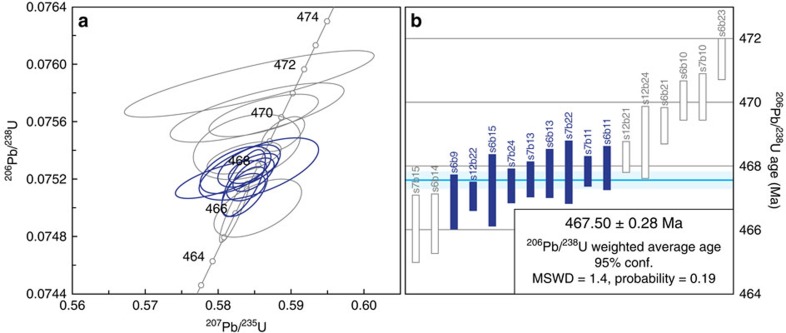
Zircon U-Pb data. (**a**) Concordia diagram, with analyses used in the age calculation indicated by blue ellipses (unused by grey). Data point error ellipses are 2*σ*. (**b**) U-Pb age distribution of analysed zircon grains, with age-defining grains indicated by blue colour. Box heights are 2*σ*.

In the course of processing 47 samples to isolate microfossils and heavy mineral grains from Kinnekulle, a single sample from the ‘Täljsten' at the Thorsberg quarry (Fig. 1) yielded an anomalous abundance (several hundred grains per kilogram of rock) of prismatic zircon. All other samples were barren of such grains. The zircon-bearing bed, locally known as the ‘Likhall' (‘Corpse slab')^12^, is visibly distinct from enclosing strata (Fig. 2a) and it is recognizable across Kinnekulle. The zircon grains are pale golden in colour and have well-defined crystal faces with no evidence of abrasion. Morphologies range from short prisms with near-equant aspect ratios to long acicular forms and crystals consistently show internal oscillatory zoning (Fig. 2b). Associated minerals include ilmenite, biotite and titanite. Among a total of 17 zircon crystals that were analysed via Chemical Abrasion Thermal Ionization Mass Spectrometry (CA-TIMS; see the ‘Methods' section), there is a clustering of analyses ∼468–467 Ma (Fig. 3; Table 1). At the core of this cluster, nine grains exhibit overlapping ^206^Pb/^238^U dates defining an error-weighted mean of 467.50±0.28 Ma (MSWD=1.4). This is interpreted as the crystallization age of the zircons and by inference the depositional age of the ‘Likhall' bed (see below). Two analyses that are slightly younger than the main cluster were excluded from the age calculation. These two grains likely fall off the main array due to a minor loss of a Pb (refs 16, 17). The remaining six grains that have older ages than the main cluster are interpreted to contain an older, perhaps xenocrystic, component. No relationship between the external characteristics of grains and their individual ages were noted.

### The timing of the L chondrite breakup event

**Figure 4:**
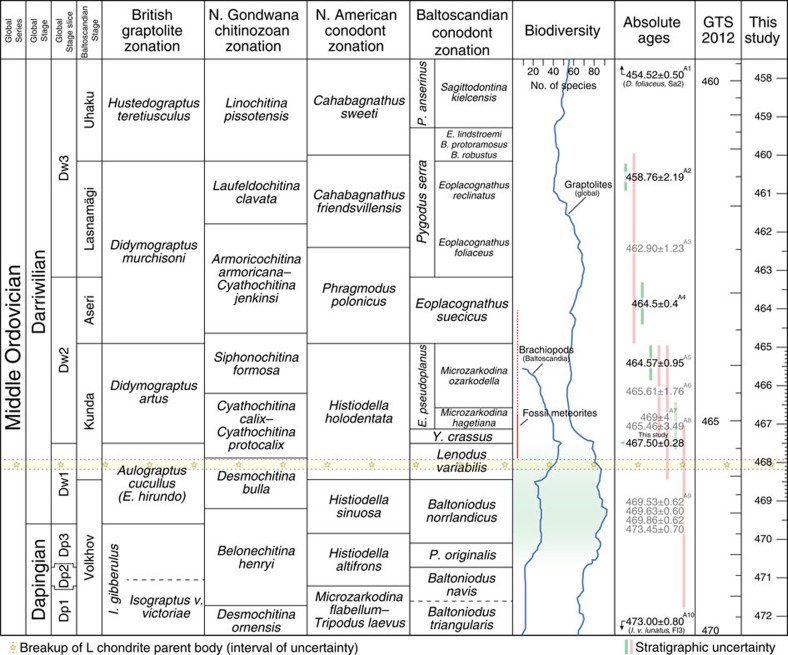
The timescale of the Middle Ordovician. A revised Middle Ordovician timescale based on our CA-TIMS data and well-constrained, high-precision datum points from the literature^13^,^27^,^58^. The resolution of tick marks in the timescale indicates confidence and precision of the calibration. GTS2012 (ref. 27) is included for comparison; differing distances between tick marks indicates the relative distortion resulting from the recalibration introduced herein. Radioisotopic dates used in the construction of the timescale are indicated by black labels, with remaining datum points in grey for reference (A1 (ref. 51); A2* (ref. 49); A3*, A6* (ref. 59); A4, A5 (ref. 23); A7 (ref. 60); A8* (ref. 61); A9 (ref. 55); A10 (ref. 50); *reported with recalculations for GTS2012 (ref. 27)). The interval corresponding to the Kunda Baltoscandian Stage is calibrated at high resolution with the aid of CRE ages of fossil meteorites. Baltoscandian brachiopod^35^,^36^ and global graptolite^38^ species diversity curves are calibrated against the biostratigraphic and geochronologic framework.

Chromite grains within fossil meteorites facilitate determination of their CRE ages via cosmogenic noble gases, which represent the time spent in space following the disruption of their parent body. Previous studies have shown that the meteorites have young ^21^Ne CRE ages (c. 0.1–1 Ma) that increase upward through the meteorite-bearing interval^18^,^19^,^20^. As such, CRE analyses provide a relative timescale spanning the meteorite-rich rock interval that translates into sedimentation rates. In combination with the numerical age obtained from the ‘Likhall' zircons, the CRE ages of fossil meteorites can be employed for geochronologic work; using the base of the short-ranged *Yangtzeplacognathus crassus* conodont Zone (see below), or lateral equivalents, as a reference level, the age of bounding strata can be deduced from the CRE data. Figure 1 illustrates this method in principle, using published CRE ages of macroscopic fossil meteorites^18^,^19^,^21^. Taken at face value, tracing of the CRE ages backward in time and stratigraphic space from the ‘Likhall' bed indicates that the meteorites were dispersed at 468.0±0.3 Ma or, in a relative reference frame, at a level c. 1 m below the ‘Täljsten' that corresponds to the early Kundan (early Darriwilian, Dw1; Fig. 1)^22^. The sedimentation rates derived from CRE ages of fossil meteorites and U-Pb dates^23^ are in excellent agreement despite a tenfold difference in both stratigraphic and temporal resolution between the two data sets. The minimum sedimentation rate implied by the CRE data (3.3 mm ka^−1^) is close to the average rate implied by U-Pb data (3.1 mm ka^−1^), but the stratigraphic distribution of fossil meteorites puts a practical lower limit on the net sedimentation rate at c. 3.8 mm ka^−1^. The maximum sedimentation rate (8.7 mm ka^−1^) calculated from the CRE data is unrealistic, as it is incompatible with the oligotrophic and sediment-starved depositional environment represented by the ‘orthoceratite limestone', and the resulting timescale is equally incompatible with the chronologic framework (Figs 1 and 4). The CRE and U-Pb data together indicate that net sedimentation rates below 5 mm ka^−1^ prevailed at Kinnekulle over longer time periods and that an average close to 4 mm ka^−1^ is appropriate.

### The Ordovician timescale

The ‘Likhall' bed is exceptionally well constrained in terms of biostratigraphy, permitting reliable correlation at the global scale. The base of the ‘Likhall' bed coincides with the boundary between the globally recognized *Lenodus variabilis* and *Yangtzeplacognathus crassus* conodont zones (Fig. 1)^13^,^24^. This is close to the boundary between the *Expansograptus hirundo* and *Holmograptus lentus* (or, *Didymograptus artus*) graptolite zones^25^,^26^ and also that between the regionally recognized *Asaphus expansus* and *Asaphus raniceps* trilobite zones^14^. Given the excellent biostratigraphic control, our new 467.50±0.28 Ma date from the ‘Likhall' bed adds a tie point to which the Ordovician timescale can be further calibrated. The date is in good agreement with bracketing datum points used for the Geologic Time Scale 2012 (GTS2012)^27^, although it implies that the base of the Darriwilian, which is presently cited as 467.3±1.1 Ma, must be moved back in time. For reference, the Dapingian–Darriwilian boundary lies at least 5 m below the base of the ‘Täljsten' at Kinnekulle. Figure 4 illustrates a revised timescale for the Middle Ordovician, based on radioisotopic data presented here and from the literature. Taking CRE and U-Pb constraints into account, a high-resolution timescale was produced for the Kunda Baltoscandian Stage using a 4 mm ka^−1^ average sedimentation rate and the stratigraphy at Kinnekulle as a model (Figs 1 and 4). In this rendition, the Middle Ordovician spans c. 457.5–472.5 Ma, as compared with c. 458.5–470 Ma in GTS2012 (ref. 27)–a 30% increase in duration.

## Discussion

Given that prismatic zircon and other heavy mineral grains commonly are either absent or very scarce (and typically anhedral/subhedral) in acid-insoluble residues from the ‘orthoceratite limestone', the abundance and nature of zircon recovered from the ‘Likhall' bed is exceptional. The angularity of the grains indicates very little or no transport within a sedimentary system and reworking was clearly limited even at the local scale (there is no ‘tailing off' in zircon abundance upward). Collectively, the evidence indicates a short-term event that entailed a significant influx of fresh zircon grains to the Kinnekulle area. Although the host bed is not recognized as a discrete bentonite horizon, we interpret the zircon grains to be the result of an ash fall related to distal active volcanism. The action of burrowing organisms, as is recorded in the ‘orthoceratite limestone' by pervasive bioturbation patterns and numerous ichnofossils^28^, would quickly mix the fallout from ash clouds into the muddy seafloor and prevent the formation of discrete bentonite beds. Still, bentonite beds do occur in the ‘orthoceratite limestone' as well as in coeval shales. Most significantly, in the province of Skåne (Scania), southernmost Sweden, a succession of bentonite beds occurs in strata coeval with the ‘Täljsten'^29^. These beds provide unambiguous evidence of active volcanism in the region. Source volcanoes were probably situated to the west or southwest (present-day directions), where magmatic activity occurred during the successive amalgamation of Baltica, Avalonia and Laurentia^30^,^31^.

The dominance of distinctly euhedral shapes and the common presence of acicular zircon grains, together with internal oscillatory zoning, indicate a magmatic origin and rapid cooling^32^, as is most typical for volcanically derived grains. The homogeneous overall appearance of the grains points towards a common origin. A single-source origin is also implied by the consistent ages of the grains. A weathering-originated detrital assemblage would likely give a range of much older ages, as any significant land areas were part of the Precambrian Baltic shield. In this regard, the distal location of the Kinnekulle area relative to the mainland during the Middle Ordovician is reflected by the complete lack of Precambrian grains in the set analysed here. As such, transport of coarser-grained and high-density detritus to the area was limited with much of the siliciclastics brought in through aeolian processes. It has even been suggested that a large proportion of the non-carbonate component of the ‘orthoceratite limestone' derives from atmospherically transported volcanic ash^33^, and that such material entailed the widespread formation of iron ooids at the time^34^.

The consistently young and well-constrained CRE ages of the lowermost occurring fossil meteorites allow for little leeway in terms of when their parent body broke up and in which stratigraphic interval one should look for signals of this event in the terrestrial sedimentary record (Fig. 1). The L chondrite breakup event and the first arrival of meteorites from it clearly post-date the onset of brachiopod diversification in Baltoscandia^35^,^36^ and Laurentia^37^, and also that of graptolites at the global scale^38^ (Fig. 4). Together, these records show that a significant diversification pulse commenced in the benthic and pelagic realms already during the Volkhovian (mid-Dapingian), some 2 Myr before the inferred asteroid breakup event. Brachiopods show accelerated diversification at the transition into the Kundan (early Darriwilian), but the onset of this phase precedes the meteorite-bearing interval. The GOBE spanned many millions of years during which different taxa show spurts in biodiversity at different times and the overall phenomenon can be described as diachronous at the global scale^5^,^9^. It has even been argued that the GOBE was initiated already during the Cambrian^8^. However, regardless of how the GOBE is constrained and defined, the refined date for the L chondrite breakup event implies that it did not initiate the biodiversification and neither paleoenvironmental proxies nor diversity patterns show any measurable influence from it. In fact, beyond the presence of meteorites, the event appears to have left no obvious record in the marine environment (occasional larger impacts notwithstanding), and the sedimentologic and paleontologic development across the meteorite-bearing interval forms a continuum with no signs of extraordinary influence or catastrophic disturbance^22^,^36^. Although these may ultimately have been astronomically forced (that is, via Milankovitch cyclicity), we advocate more conventional processes, such as climate change, plate tectonics and ecologic feedback, as drivers behind the biodiversification during the GOBE^15^,^36^,^39^.

Sediment-dispersed L-chondritic chromite holds promise to be used as a unique tool for the construction of empirically based high-resolution (kyr) timescales globally. Age dating via CRE analyses can be applied to individual meteoritic chromite grains, which occur abundantly in Middle Ordovician strata formed after the breakup of the L chondrite parent body^2^. The results of Meier *et al*.^20^ wherein Swedish and Russian strata were successfully correlated and tested against biostratigraphy using CRE data from sediment-dispersed L-chondritic chromite, validate this method. However, as most of the grains appear to represent micrometeorites, methods to eliminate the common overprint of a solar wind signal within the noble-gas content should be employed. Sediment-dispersed L-chondritic chromite can further be used as an independent correlation tool without CRE analyses, as the first arrival of such grains in the wake of the parent body breakup event essentially resulted in a chronohorizon in the sedimentary record. An enhanced influx of L-chondritic meteorites appears to have persisted well into the Darriwilian, at least until the late *Eoplacognathus suecicus* conodont biochron (∼463.5 Ma)^23^,^40^ and, thus, there is potential to work out the detailed geochronology for this interval using CRE analyses of sediment-dispersed chromite. Such an endeavour may reveal additional beds enriched in volcanic zircon that would provide CA-TIMS dates for calibration of the CRE chronometry. Cyclostratigraphic analyses of paleontologic and sedimentologic data may be utilized to further optimize timescales. The results will yield important empiric information about the dynamics of asteroid collisions (for example, cascading effects) and the influx of ordinary chondritic matter to Earth through time. L chondrites remain common in the meteoritic influx to Earth even today, but their CRE ages rarely exceed a few tens of million years^1^,^41^. Clusters of CRE ages suggest that the sedimentary record should host several pulses of L chondrite bombardment and the Middle Ordovician fossil meteorites appear to represent only the first record of such an event yet known.

## Methods

### Separation and search for zircon grains

The acid-insoluble residues of 47 (69 incl. replicates) variably sized (c. 0.5–10 kg) samples from the ‘orthoceratite limestone' at Kinnekulle, stored at the Department of Geology, Lund University, were carefully searched for >63 μm heavy mineral grains. Depending on size and original purpose, the limestone samples were processed via different acid-treatment protocols^42^,^43^ (typically using acetic acid followed by formic acid for smaller samples, hydrochloric acid followed by hydrofluoric acid for larger samples). The sample series spans the upper Volkhov through topmost Kunda Baltoscandian stages, with the ‘Täljsten' and its enclosing strata covered at bed-by-bed resolution by several replicate samples (Fig. 1).

### U-Pb analyses

A total of 17 zircon grains from the ‘Likhall' bed, with varying morphological characteristics, were selected for U-Pb dating using the CA-TIMS^44^ method. Analyses were performed at the Centre for Star and Planet Formation (StarPlan), at the Natural History Museum of Denmark, University of Copenhagen, Denmark. The grains were chemically abraded to remove damaged domains and minimize the effects of Pb loss. This pre-treatment consisted of thermal annealing of all crystals for 3 days at 900 °C in Alsint crucibles. Un-annealed domains were then dissolved using concentrated HF for 12 h at 180 °C, in Savillex beakers placed inside vapour equilibrated Parr bombs. Before complete dissolution, the zircon grains were cleaned in alternating steps with warm 3.5 M HNO_3_, H_2_O and acetone. They were processed as single grains to account for the possible existence of optically indistinguishable xenocrystic grains. As such, the crystals were individually dissolved in Teflon capsules in an HF–HNO_3_ (3:1) mixture, together with the mixed ^202^Pb–^205^Pb–^233^U–^235^U EARTHTIME U-Pb tracer, for 5 days at 210 °C. The dissolved samples were dried down and redissolved in 3 M HCl overnight, and then dried down again with 8 μl of 0.1 M H_3_PO_4_. They were loaded with silica gel^45^ on zone-refined rhenium single filaments. The Pb and U isotopic ratios of the sample-tracer mixture were measured using a Thermo Fisher thermal ionising mass spectrometer, where each isotope was sequentially counted in a single axial ion counting system with Pb as Pb^+^, and U as UO^2+^. All common Pb was considered as procedure blank.

The data correction, reduction and age calculation was accomplished using an Excel spreadsheet and ISOPLOT^46^. A linear mass fractionation normalization for instrumental mass-dependent fractionation of Pb was based on the ^202^Pb/^205^Pb isotopic ratio of the tracer. Mass-dependent fractionation during U isotope measurement was corrected by running U standards and the isobaric interference of ^233^U^16^O^18^O on the ^235^U^16^O_2_ peak at mass 267 was also corrected. The ^238^U and ^235^U decay constants of Jaffey *et al*.^47^ and a ^238^U/^235^U ratio of 137.88 were used for the fractionation and age calculations. All age uncertainties are quoted at the 95% confidence level.

### Cosmic-ray exposure ages of fossil meteorites

The data on CRE (^21^Ne) ages of fossil meteorites in the studied succession derive from Heck *et al*.^18^,^19^ and Schmitz *et al*.^21^. The data points were spaced according to stratigraphic distance, based on the assumption that the meteorites were found in the middle of their host bed (many meteorites lack detailed data in this regard). The extreme lines of sedimentation rate/time development (CRE Max. and CRE Min. in Fig. 1) were determined from maximum inclinations possible via direct connection of central age data points from the lowermost-lying meteorite level. While somewhat simplified in a statistical sense, changes in parameters (for example, stratigraphic position of meteorites, CRE ages) within realistic constraints have little effect on the main outcome and subsequent interpretations. The effects of varying statistical parameters on the intercept (‘year 0') level in the CRE data were however taken into account in the definition of the stratigraphic interval that coincides with the initial dispersion of the fossil meteorites that we correlate with the breakup of their parent body (Fig. 1). Bracketing CRE ages indicate that the zircon-bearing ‘Likhall' bed formed c. 0.4±0.15 Ma after the disruption of the L-chondrite parent body, or relative to a level c. 1 m below the base of the ‘Täljsten'. Interpolation further reveals that the bed itself represents a time period of 10–40 ka. The uncertainty associated with the CA-TIMS age thus appears to be close to the stratigraphic resolution of the studied strata.

Meteorite ages in the upper part of the studied succession lying towards the younger end of the CRE diagram probably stem from unusually large host bodies, that provided sheltering from cosmic rays during the voyage through space^18^. The faded CRE data points in Fig. 1 (Gull 001 and Gla 3 003) were not considered in the data interpretation. The stratigraphic position of Gull 001, which was found in a different quarry than the other meteorites were found, is not known in any detail beyond biozone (*Yangtzeplacognathus crassus*)^48^ and the relationship of the non-L-chondritic Gla 3 003 (ref. 21; Österplana 65)^4^ to the main meteorite population found at the Thorsberg quarry is not clear. Nevertheless, the CRE age of Gull 001 fits well into its stratigraphic context in relation to the meteorites from Thorsberg (as does the age of Gla 3 003).

### Sedimentation rate

The lowermost occurrence of fossil meteorites and abundant L-chondritic chromite puts a lower limit on the possible net sedimentation rate throughout the studied succession at c. 3.8 mm ka^−1^ with the conditions set by the CRE data as above. The upper limit of the net sedimentation rate in the studied succession is more difficult to define, but the U-Pb data indicates c. 4.6 mm ka^−1^ if we include the calculated error of our CA-TIMS age (467.50±0.28) and a stratigraphic uncertainty of the overlying datum point (464.57±0.95 Ma)^23^ corresponding to 25% of the Kunda Baltoscandian Stage. From this, we can constrain the average sedimentation rate of the Kundan to c. 4.2±0.4 mm ka^−1^. Taking into account perceived variations in sedimentation rate across this interval, a net sedimentation rate of 4 mm ka^−1^ is deemed realistic.

### Constructing a timescale

The basic timescale in Fig. 4 was produced by an initial linear interpolation between the central dates of U-Pb datum points^23^,^49^,^50^,^51^ and fitting of these onto the stratigraphic framework of the Geologic Time Scale 2012 (GTS2012)^27^. Where possible, the timescale was then calibrated on a point-by-point basis with focus on boundary-defining dates. In instances where dates overlap in error, the data were treated as a single time span of which the mid-point of the middle third portion defines the boundary and the midpoints of the bounding thirds define the ‘actual' numerical ages of bracketing datum points. Any part of the error span of the lower-lying datum point passing the minimum age defined by an overlying point was disregarded (a lower-lying point cannot be younger than an overlying one). Biostratigraphy was treated as secondary to numerical dates. Boundaries without defining bracketing dates were left in their relative position in the biostratigraphic scheme used in GTS2012 (ref. 27).

The Kunda Baltoscandian Stage was further calibrated using CRE ages of fossil meteorites, and a high-resolution timescale (4 mm ka^−1^ scale in Fig. 1) was produced for this time span. The timescale in the Dapingian was roughly defined through extrapolation of the CRE-derived scale, as directly applied to the stratigraphic succession at Kinnekulle^22^,^52^,^53^,^54^. Given the dearth of well-constrained datum points in this interval, the base of the Dapingian was arbitrarily adjusted to 472.5 Ma to allow for a realistic amount of time relative to the central date of the underlying datum point (473.0±0.8 Ma, uppermost Floian *Isograptus victoriae lunatus* graptolite Zone)^50^. For reference, the extrapolation resulted in an age of c. 472.85 Ma for this level. The large stratigraphic distance between bracketing datum points, coupled with the poor temporal constraint of the lower one of these, results in a large uncertainty for the Middle-Upper Ordovician (Darriwilian-Sandbian) boundary (Fig. 3). Simple linear interpolation yields an age of c. 457.5 Ma. Determining a robust numerical estimate of the true uncertainty associated with this boundary age is difficult, as the bracketing datum points with uncertainties form a time span of nearly 7 Ma.

Only biostratigraphically well constrained and high-resolution (high analytical precision) datum points associated with U-Pb data were included in the construction of the timescale. Many of the points used in GTS2012 (ref. 27) were excluded due to a lack of biostratigraphic and/or analytical precision. Likewise, although the dates are quite precise, we disregarded the bentonite data of Thompson *et al*.^55^ due to a significant lack of biostratigraphic control^56^. We do note, however, that their data appears to substantiate a revision of the Middle Ordovician timescale as proposed here, as it suggests that 469 Ma is reached already in the Dapingian–Darriwilian boundary interval^57^ (cf. GTS2012 (ref. 27)). In total, six datum points (excluding CRE data) were used for the calibration of the timescale. While this number is essentially identical to that used in the GTS2012 for the same interval^27^, the datum points used here are typically better defined both in terms of biostratigraphic and chronometric precision.

### Data availability

The authors declare that data supporting the findings of this study are available within the article and, where applicable, in cited literature.

## Additional information

**How to cite this article:** Lindskog, A. *et al*. Refined Ordovician timescale reveals no link between asteroid breakup and biodiversification. *Nat. Commun.* **8**, 14066 doi: 10.1038/ncomms14066 (2017).

**Publisher's note:** Springer Nature remains neutral with regard to jurisdictional claims in published maps and institutional affiliations.

## Figures and Tables

**Table 1:**
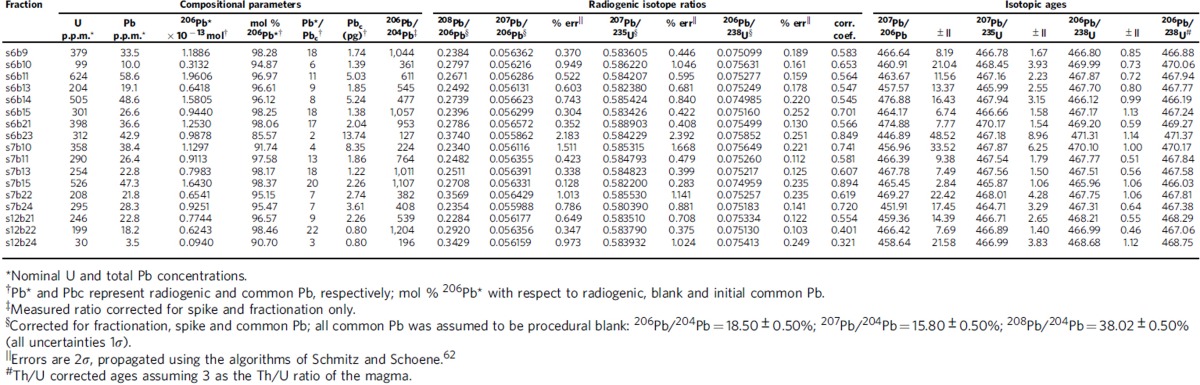
U-Pb isotopic data from the ‘Likhall' zircons.
